# Si-Ho Tchou: life of a legend from physiology to psychology

**DOI:** 10.1007/s13238-018-0553-4

**Published:** 2018-05-21

**Authors:** Wei Chen, Xi Chen, Shengjun Wen

**Affiliations:** 1 Department of Psychology, Shaoxing University, Shaoxing, 312000 China; 2 Department of Psychology, The Education University of Hong Kong, Hongkong, China; 3 College of Humanities, Shanghai University of Finance and Economics, Shanghai, 200433 China; 4 Department of Cognitive Neurology, Hertie Institute for Clinical Brain Research, University of Tübingen, 72076 Tübingen, Germany

*Why do the bees renounce sleep*,* the joys of honey*,* love*,* and divine leisure*?* Why do they get so much pain and effort*,* and where does such a decisiveness come from*?* So the sex they are dying for must deserve this sacrifice*,* it must be more beautiful*,* happier*,* and do something they can not do*?

——Maurice Maeterlinck, Das Leben der Bienen, 1901

**Figure 1:**
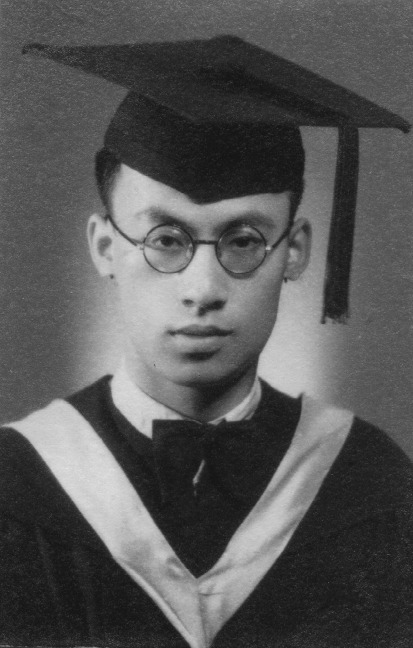
Si-Ho Tchou in 1937

Dr. Si-Ho Tchou (朱锡侯, 1914–2000) was born in the northeast part of Jilin Province, although his ancestry can be traced to Shaoxing, Zhejiang Province (Fig. 1). Dr. Tchou graduated from the L’Institut Franco-Chinois de Lyon (IFCL) in 1937 and was selected to continue his studies in France. Dr. Tchou spent eight years in France, where he first obtained his PhD in physiology from the University of Lyon and then received his PhD in psychology from the University of Paris. Dr. Tchou returned back to China in 1945 and was appointed as a professor in physiology at Yunnan University School of Medicine. Meantime, he was also appointed as a professor in psychology and aesthetics at Yunnan University, School of Humanities and Law. In 1980, he became the professor of physiology and psychology at the department of psychology in Hangzhou University. Then he served as a member of the physiological psychology committee in Chinese Psychological Society.

**Figure 2:**
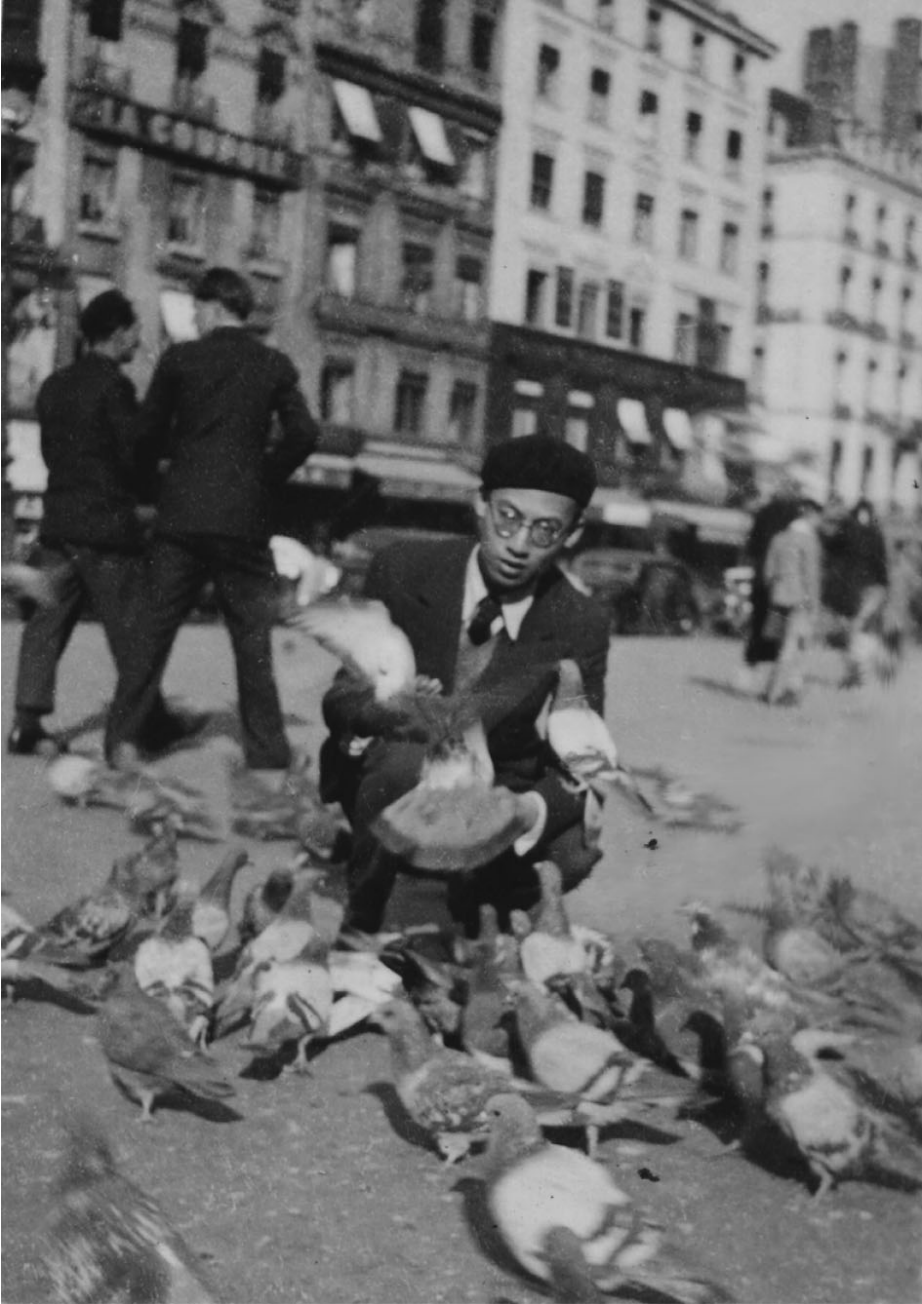
Si-Ho Tchou in Lyon

Dr. Tchou has made significant achievements during the eight years’ study in France. At the University of Lyon, Dr. Tchou first studied aesthetics and psychology under the guidance of the famous aesthetician and psychologist Prof. Étienne Souriau and received master’s degree in Arts (Fig. 2). Then he transferred to Medical School to study under the supervision of a brilliant physiologist Prof. Henri Cardot. He is also the student of Prof. Charle Richet who was the Nobel Laureate in physiology or Medicine in 1913. Both of them are well known for their researches on the body’s immune reactions to foreign substances (Stewart, 2012).

During the holidays, Dr. Tchou joined his mentor Prof. Cardot to the marine life station by the Mediterranean Sea, where he conducted researches on animal nerve cells and nerve fibers, spending days and nights recording nerve impulses in a tiny laboratory. At that time, their researches focused on the glandular giant neurons of marine invertebrates like *Aplysia*, and they had to go daily to the sea to capture *Aplysia*. Dr. Tchou was responsible to differentiate the glands and giant neurons of *Aplysia* based to their weight, and the other research project he conducted was to compare the neural discharge rhythms of *Aplysia*, based on their weight. At the end of 1942, Dr. Tchou was awarded a PhD degree in Science from the University of Lyon for his work on the growth pattern and rhythmic discharge phenomenon of neurons in *Aplysia*. His findings showed that the glands and giant neurons of *Aplysia* could be easily identified and sorted out. In addition, he found that the cell size was only a superficial feature of cell differentiation (Tauc, 1966; Ingoglia and Sturman, 1980; Ambron and Kremzner, 1982; Abel and Kandel, 1998).

As early as 1942, Dr. Tchou has studied the giant neurons of *Aplysia*, and together with Dr. Arvanitaki, published the paper “Laws of the relative individual growth of nerve cells in *Aplysia*”, where they recorded and quantified the number and relative size of individual neurons at different stages (Arvanitaki and Tchou, 1942). Based on this work, French scientists Dr. Tauc (1954) and Dr. Arvanitaki (1955) pioneered researches on single cell recording of giant neurons using microelectrodes. In addition, benefiting from the methods reported by Dr. Tchou, Prof. Kandel’s team found that habituation and dishabituation was due to the synaptic potential of gill motor neurons (Pinsker et al., 1970; Kupfermann et al., 1970; Carew and Kandel, 1973; Castellucci and Kandel, 1976; Kandel and Schwartz, 1982; Voronezhskaya and Croll, 2015).

Scientists continued to conduct in-depth researches in *Aplysia* and made numerous significant research findings, however, as pointed out by Hughes (1968): “*apart from the work of Tchou Si-Ho* (*Tchou*, 1942) *and Arvanitaki few quantitative observations seem to have been recorded on the relative size of its nervous system or on the numbers and sizes of neurons in individuals of different sizes* (p. 423)”. Thus, Dr. Arvanitaki and Dr. Tchou Si-Ho were the first ones who studied this feature in the abdominal ganglion of *Aplysia punctata* (Hughes and Tauc, 1961; Kandel and Tauc, 1965; Koester and Kandel, 1977; Adams and Benson, 1985; Bailey and Chen, 1988; Cleary et al., 1991). As neurons are very difficult to grow, neurobiologists heavily depended on the established research methods, and Dr. Tchou’s work on the quantification of neuron cells and rhythmic discharge in *Aplysia*, as well as the experimental methods established during the researches, laid a solid foundation for the studies using *Aplysia* as a model organism. The following researches by many prominent neuroscientists has benefited from Dr. Tchou’s pioneering work. Such as Prof. Eric R. Kandel at Columbia University, who found that memory was linked to the synapses that connect nerve cells and uncovered its molecular mechanisms in *Aplysia*. Prof. Eric Kandel was awarded the Nobel Prize in Physiology or Medicine in 2000. This was inseparable from the foundational work of Dr. Tchou and his colleagues on the investigation and establishment of *Aplysia* as a model organism (Coggeshall et al., 1966; Hughes, 1968;  Peretz and Lukowiak, 1975; Shapiro et al., 1980).

Dr. Tchou had great interests in physiology, especially experimental psychology. Thus, from 1943 to 1945, Dr. Tchou enrolled in the University of Paris (Sorbonne), and began his researches on physiological psychology and applied psychology under the supervision of Profs. Paul Fraisse and Rene Zazzo, and later with the central figure of French psychology in the twentieth century, Prof. Henri Piéron, who was not only well known for his research in sensory physiology, but also the founder and director of the Institute of Psychology at the University of Paris. With the profound influence of Prof. Piéron, Dr. Tchou embarked on researches in physiological psychology and studied changes to various nerve cell activities in the brain resulting from human psychological activities in order to uncover the mystery origin of human life. In July 1945, Dr. Tchou decided to give up the plan to finish the PhD thesis in physiological psychology in Prof. Piéron’s laboratory, rejected the invitation of Prof. Alfred Burloud to stay at Rennes University and returned back to China.

**Figure 3:**
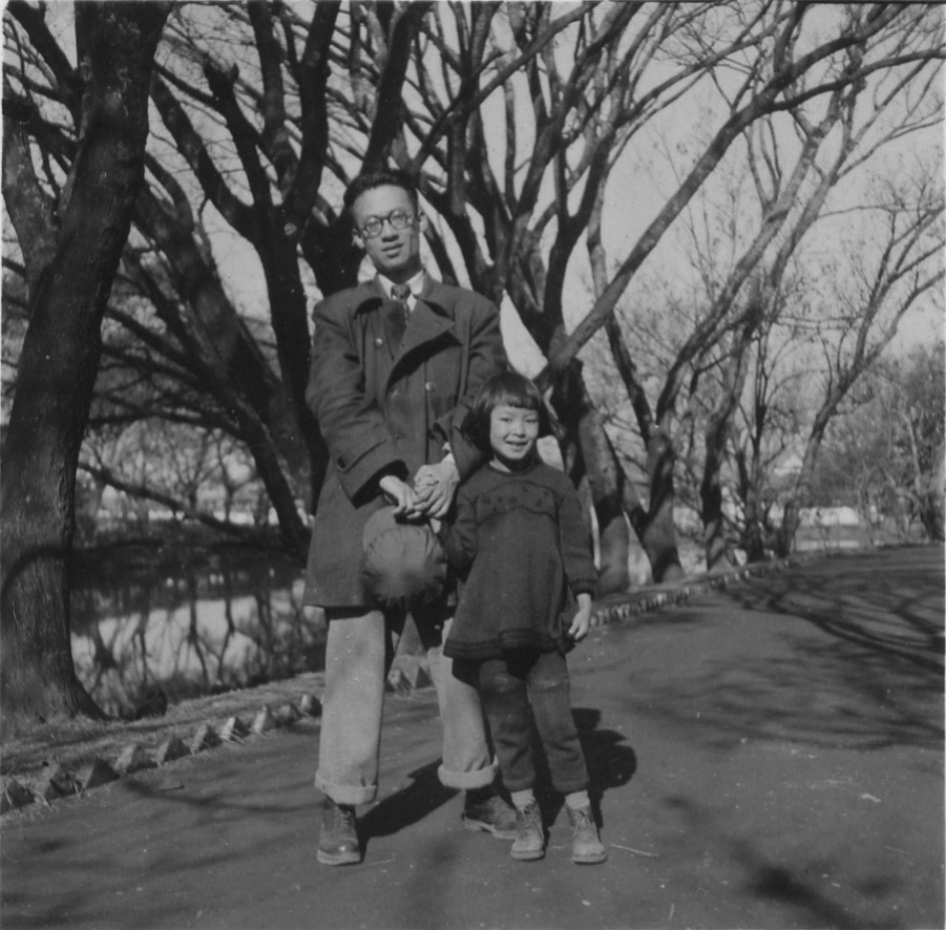
Si-Ho Tchou with his eldest daughter in Yunnan University, circa 1955

In the autumn of 1945, Dr. Tchou accepted the offer from Hiong King-lai, the President of Yunnan University. From 1945 to 1957, Dr. Tchou worked hard at Yunnan University in spite of the poor teaching conditions and the difficulties he faced in his work and life (Fig. 3). With the belief of saving the nation with science and education, he started his career under the harsh conditions. With the lack of teaching materials and laboratory equipments, Dr. Tchou spent a lot of time and energy translating and writing teaching materials and paid out of his own pocket for the laboratory equipments. Finally, Dr. Tchou established the department of physiology at Yunnan University School of Medicine, introduced scientific research equipments, and set up technical rooms and observation rooms. There, he conducted researches on “pain and skin reflex”, “functional adaptation after anastomosis of the intestines and the ureter and bladder”, and “the effect of acupuncture stimulation of Zusanli on digestive hematopoietic function”.

**Figure 4:**
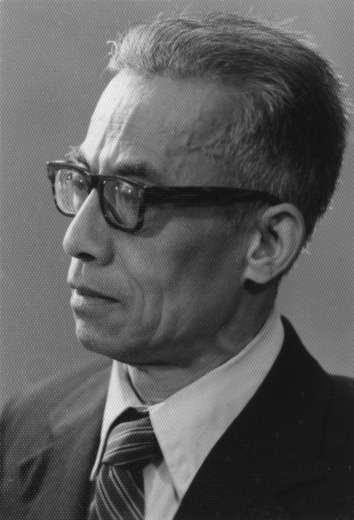
Si-Ho Tchou in the early to the Hangzhou University

Unfortunately, Dr. Tchou was innocently involved in a political turmoil in 1957. After experiencing major hardships in life, Dr. Tchou went to Hangzhou University (Fig. 4). “To be honest, I am already 66 years old. I really hope to be able to return to psychology in my remaining years and do my bit for education in China… I just want to work… (朱锡侯, 2011, p. 197)”. At that time, psychology has been neglected in China for decades and was treated as a pseudoscience of the bourgeoisie. There was an enormous gap with the international community in this research area. Although the setting up of physiological psychology classes has been proposed, there were no references available domestically. Therefore, Dr. Tchou searched through a large number of foreign texts and magazines, excerpted the information that was suitable for the physiological psychology classes and translated some of the basic teaching materials such as *Introduction to Physiological Psychology* by Allen Schneider and Barry Tarshis; *Introduction to Physiological Psychology* by Charles Levinthal. On November 20, 1980, the inaugural assembly and symposium of the physiological psychology professional committee of Chinese Psychological Society was held in Nanjing Normal University, and as one of the organizing committee members, the two textbooks translated by Dr. Tchou were also included in the proceedings. Indeed, Dr. Tchou has paved the way for the development of physiological psychology and was certainly the pioneer of physiological psychology. While the teaching conditions were tough, it allowed him to develop a close relationship with his students. He often directed student essays, and discovered a constant relationship between time perception and the adjustment of knowledge and experience (朱琪, 1985).

Dr. Tchou made brilliant achievements in the fields of Western philosophy, aesthetics, physiology and psychology. With his in-depth researches in understanding the mysteries of human brain functions, he made outstanding contributions to the development of physiology and psychology in China. As he once said, “There is a common thread running through my lifetime’s learning, that is, to know the mystery of life, to understand people’s inner worlds, their psychological activities and psychological lives… It is the logical connection in my journey beginning from philosophy, to psychology, aesthetics, neurophysiology, physiological psychology, and finally, the life sciences (朱锡侯, 2011, p. 210)”.
